# Isogenic Japonica Rice Koshihikari Integrated with Late Flowering Gene *Hd16* and Semidwarfing Gene *sd1* to Prevent High Temperature Maturation and Lodging by Typhoon

**DOI:** 10.3390/life12081237

**Published:** 2022-08-15

**Authors:** Motonori Tomita, Ryotaro Tokuyama

**Affiliations:** Research Institute of Green Science and Technology, Shizuoka University, 836 Ohya, Suruga-ku, Shizuoka City 422-8529, Shizuoka, Japan

**Keywords:** rice, late flowering gene, semidwarf gene, isogenic Koshihikari, WGS, *Hd16*, *sd1*, co-integration, new green evolution

## Abstract

We developed semidwarf and late-maturing isogenics of Koshihikari to stabilize high yield and avoid high temperature maturation. Whole-genome analysis (WGS) was conducted to examine the transitional changes in the entire genome, the size of DNA fragments integrated with the target gene, and genes accompanying the target gene owing to the progress of backcrossing. In both Koshihikari Hd16 (BC_7_F_4_) and Koshihikari sd1Hd16 (BC_8_F_2_), an SNP from adenine to guanine was detected in *Hd16* at 32,996,608 bp on chromosome 3, which is known to be a causative mutation of *Hd16* in Nipponbare. In Koshihikari sd1Hd16 (BC_8_F_2_), an SNP from thymine to guanine was detected in *sd1* at 38,267,510 bp on chromosome 1. From BC_7_ to BC_8_, the size of the DNA fragment integrated with *Hd16* decreased by 5871 bp. Koshihikari sd1Hd16 flowered 12.1 days later than Koshishikari or Koshihikari sd1 did and was 14.2 cm (15%) shorter than Koshihikari. The yield in Koshishikari sd1Hd16 (63.2 kg/a) was 7.0% higher than that of Koshihikari. This is a new germplasm designed to avoid heat damage at ripening during high-temperature summer periods by late maturation owing to *Hd16* as well as to avoid lodging by autumn typhoons by semidwarfness owing to *sd1*.

## 1. Introduction

Climate change because of global warming causes damage to crops globally [1,2]. In Japan, disastrous rainfall and floods, such as the “Heavy Rain in July, Heisei 30” [3], and large typhoons with wind speeds over 54 m/s, including Jebi and Trami, which were comparable to the worst typhoon in Japan’s history (Isewan Typhoon) [4], have been occurring frequently every year [3,4,5,6,7]. These extreme weather phenomena have caused marked damage to agriculture, forestry, and fisheries (totaling 436.5 billion yen) [8]. Under these climate crises, rice must be robust and resistant to lodging [2,9].

A moderate reduction in crop height, namely, semidwarfism, improves lodging resistance to wind and rain at the full-ripe stage, light-interception properties, and nitrogen responsiveness [10]. The development of rice semidwarf varieties enhanced their adaptability to heavy manuring and markedly increased global rice productivity by up to double between 1960 and 1990 [11]. This has been referred to as the “Green Revolution”, and is considered as the greatest agricultural contribution in the history of humankind. The gene contributing to “Green Revolution” in rice was identified as *sd1*.The *sd1* alleles, on the long arm of chromosome 1 [12,13,14], encode a defective C20-oxidase in the gibberellin (GA) biosynthesis pathway (GA 20-oxidase, OsGA20ox2) [15,16,17] and mutations in the GA20-oxidase gene lead to disruptions at a late stage of the GA pathway [15]. No detrimental effect on grain yield are conferred by *sd1* [18,19,20].

The japonica rice Koshihikari is a leading variety in Japan, accounting for 33.7% of rice acreage in the country. Koshihikari is globally valued and produced, including in the United States and Australia. However, Koshihikari suffers considerably from severe lodging damage owing to frequent heavy rains, floods, and strong typhoons; thus, the development of lodging-resistant Koshihikari has been a longstanding challenge. The first author introgressed the semidwarf gene *sd1* from Jukkoku to Koshihikari by backcrossing to Koshihikari eight times to develop a semidwarf form of Koshihikari, which was approximately 20 cm shorter than Koshihikari [21] and consisted of more than 99.8% of the Koshihikari genome, except for *sd1* derived from Jukkoku [21,22]. This cultivar was named Hikarishinseiki (rice cultivar number 12273) [21,23]. Hikarishinseiki was the first cultivar of semidwarf isogenic Koshihikari, with *sd1* registered in Japan and the United States [24,25].

Koshihikari also suffers from poor filling and widespread yield reduction because of high temperatures caused by heat waves. If the average daily temperature exceeds 23–24 °C during the 20 days after heading, a white immature grain arises [26,27,28,29], both white-back immature grains and milky-white immature grains arise at 27 °C, white-back immature grains occur at 30 °C, and milky-white immature grains frequently occur at 33 °C [30]. Recent heat waves caused 170,000 tons of high-temperature damage, namely, deterioration in rice quality, which accounted for up to 21% of the total production volume [31]. This is because the leading variety, Koshihikari, comprises 33.7% of rice acreage in Japan [32] and flowers and ripens in the high-temperature phase in August. In 2010, when the average temperature in August was approximately 2.25 °C higher than the yearly average, the grain quality noticeably degraded, and the proportion of 1st-grade rice was 23.1% lower than in 2009 [33]. To maintain the commercial status of Koshihikari, avoiding heading and ripening during the high-temperature phase through genetic modification is necessary. Rice industries urgently require late-maturing varieties instead of Koshihikari to avoid high-temperature ripening. Genetic modifications in day length responsiveness enable the alteration of rice maturation to the early or late phases, and to extend regional adaptability of genotypes and dilute the current overconcentration of Koshihikari.

A Japanese native species, Isehikari, was discovered in 1989 in the rice paddy fields of Ise Jingu Shrine as the only standing mutant among the entirely lodged Koshihikari plants after a typhoon [34,35]. This cultivar can withstand typhoon wind speeds exceeding 50 m/s and matures 10 days later than Koshihikari [36,37]. Isehikari demonstrated a high yield of 700 kg under the “Environmental Future City Concept” project of Yamaguchi Prefecture [38]. Thus, Isehikari is an extremely beneficial genetic source for late maturation and high yield. In a previous study, we identified a gene for late maturity of Isehikari and developed a Koshihikari-type isogenic line that matured 12 days later, via continuous backcrossing to a recurrent parent Koshishikari using the latest maturing segregant in F_2_ of Koshihikari × Isehikari as a non-recurrent parent [39]. Through the process of backcrossing, the 12-day later maturity derived from Isehikari was inherited as a single gene in the genetic background of Koshihikari. Finally, whole-genome analysis revealed that the late-maturing gene of Isehiakri is *Hd16*. In this study, we developed semidwarf and late-maturing isogenics of Koshihikari for the purpose of stabilizing high yield and avoiding high temperature maturation.

Through the development of isogenic lines, the proportion of the genome of the recurrent parent theoretically increased to 96.9, 98.4, and 99.2% by backcrossing 4, 5, and 6 times, respectively. However, whether this change occurs in the entire genome or around the target gene is not known. In this study, we examined backcrossed isogenic BC_7_ to BC_8_ generations in Koshihikari, the semidwarf gene *sd1*, and the late-maturing gene *Hd16.* Whole-genome analysis was conducted to examine transitional changes in the entire genome, the size of DNA fragments integrated with the target gene, and genes associated with the target gene owing to the progress of backcrossing.

## 2. Materials and Methods

### 2.1. Development of Koshihikari sd1Hd16

**Figure 1:**
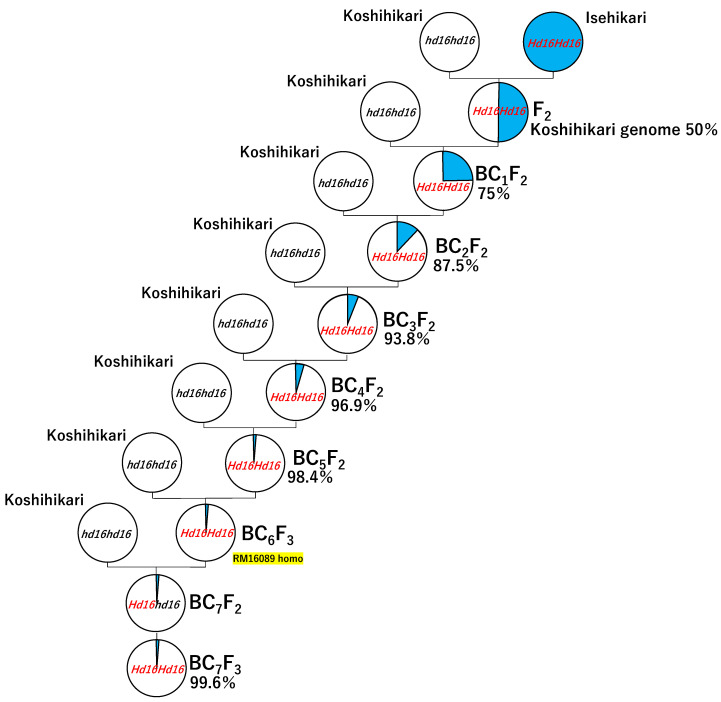
Phylogenetic process of Koshihikari Hd16 (Koshihikari × 7/[(Koshihikari × Isehikari) F_2_ late-maturing type] BC_7_F_3_).

Koshihikari Hd16 is a late-maturing Koshihikari-type isogenic line developed by integrating the 2 weeks late-maturing gene (*Hd16*) derived from Isehikari with the genetic background of Koshihikari via six continuous backcrosses to a recurrent parent Koshihikari using a late-maturing segregant in F_2_ of Koshihikari × Isehikari segregated as a non-recurrent parent [40]. In the present study, the seventh backcross with Koshihikari was conducted using Koshihikari Hd16 (BC_6_F_3_) as the pollen parent, and the Koshihikari × 7/[(Koshihikari × Isehikari) F_2_] BC_7_F_2_ plants were tested (Figure 1). The *Hd16* homozygous plant (BC_7_F_2_) was selected by gene diagnosis using the single sequence repeat (SSR) marker RM16089, and BC_7_F_3_, the BC_7_F_4_ progeny of the *Hd16* homozygous plant (BC_7_F_2_) was tested. For all plants, we investigated ear emergence day, culm length, and morphological traits and conducted gene diagnosis of *Hd16* using RM16089.

**Figure 2:**
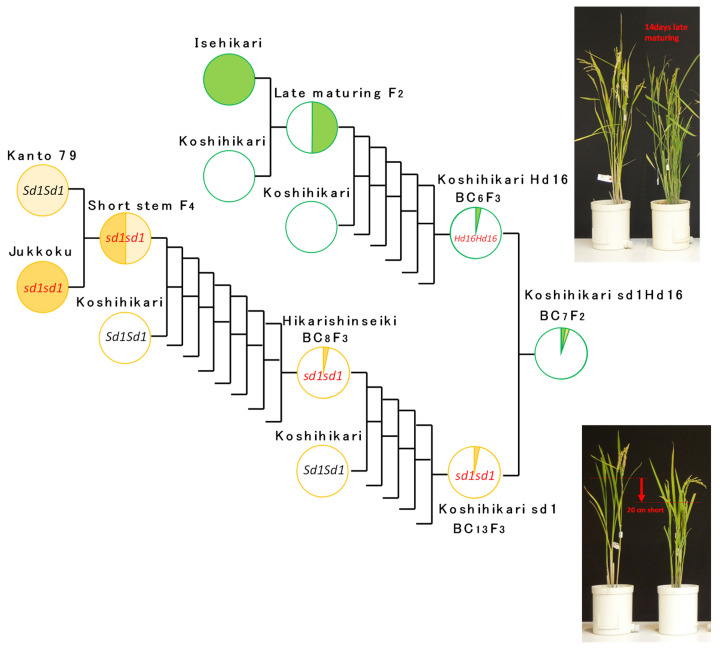
Phylogenetic process of Koshihikari sd1Hd16 (Koshihikari/Koshihikari *sd1*/Koshihikari × 6/[(Koshihikari × Isehikari) F_2_ late-maturing type] BC_7_F_2_).

Koshihikari sd1 was crossed with the late-maturing Koshihikari-type isogenic gene line (Koshihikari Hd16 (BC_6_F_2_)), and the F_2_ (equivalent to BC_7_F_2_) was tested to develop a semidwarf late-maturing Koshihikari-type isogenic line (Koshihikari sd1Hd16), which combines the semidwarf gene *sd1* and the late-maturing gene *Hd16* (Figure 2). For the *sd1* allele, the genotype was determined by culm length, and the genotype of *Hd16* was determined using RM16089 near the *Hd16* allele. The eighth backcrossing to Koshihikari was conducted using the *Hd16Hd16Sd1sd1* plant (BC_7_F_2_) segregated in BC_7_F_2_, and the Koshihikari/Koshihikari sd1/Koshihikari × 6/[(Koshihikari × Isehikari) F_2_] BC_8_F_2_ generation was tested. The ear emergence day, culm length, and morphology were investigated, and genetic diagnosis was conducted using RM16089. Whole genome analysis by next-generation sequencing was conducted for Koshihikari Hd16 (BC_7_F_4_) and Koshihikari sd1Hd16 (BC_8_F_2_).

Genetic material cultivation was conducted in a paddy field at Shizuoka University, Shizuoka, Japan, from 2013 to 2021. Genetic material BC_n_F_1_ was grown from April to July, and BC_n_F_2_ was grown from July to November. In other words, we accelerated the generation in a short period. Finally, the obtained genotypes were grown from May to October to test performance. Seedlings were individually transplanted into a paddy field in mid-July at a transplanting density of 22.2 seedlings/m^2^ (one seedling per 30 × 15 cm). The paddy field was fertilized with 4.0 kg of basal fertilizer containing nitrogen, phosphorus, and potassium (weight ratio, nitrogen:phosphorus:potassium = 2.6:3.2:2.6), with 4.3 g/m^2^ nitrogen, 5.3 g/m^2^ phosphorus, and 4.3 g/m^2^ potassium across the field. The heading date was recorded as the date on which the first panicle emerged from the flag leaf sheath for each plant. Culm length was measured as the length between the ground surface and the panicle base. For the yield test, after ripening, 10 plants typical of each genotype were sampled twice. The sampled plants were air-dried and assessed or measured for the following traits: panicle length, number of panicles, number of florets/panicles, proportion of fertile florets, total panicle number, and weight of unmilled rice/1000 grain. The yield of unpolished rice was calculated using the following equation: Yield of unmilled rice (g/m^2^) = (number of panicles/m^2^) × (number of florets/panicle) × (proportion of fertile florets) × (weight of unmilled rice/grain). Lodging degree was determined based on the inclination angle of plant; 0: standing, 1: almost 70, 2: almost 50, 3: almost 30, 4: almost 10, 5: lodged. Taste evaluation was based on a seven grade-organoleptic assessment by a panelist, and protein contents were determined using Infratec 1241(VOSS Japan Ltd., Tokyo, Japan). The means of traits were statistically compared using the *t*-test.

Koshihikari Hd16 is a late-maturing Koshihikari-type isogenic line developed by integrating the 2 weeks late-maturing gene (*Hd16*) derived from Isehikari with the genetic background of Koshihikari via six continuous backcrosses to a recurrent parent Koshihikari using a late-maturing segregant in F_2_ of Koshihikari × Isehikari segregated as a non-recurrent parent.

Koshihikari sd1 was crossed with a late-maturing Koshihikari-type isogenic gene line (Koshihikari Hd16 (BC_6_F_2_)) to develop a semidwarf late-maturing Koshihikari-type isogenic line (Koshihikari sd1Hd16), which combines the semidwarf gene *sd1* and the late-maturing gene *Hd16*.

### 2.2. Whole Genome Sequence Analysis

**Figure 3:**
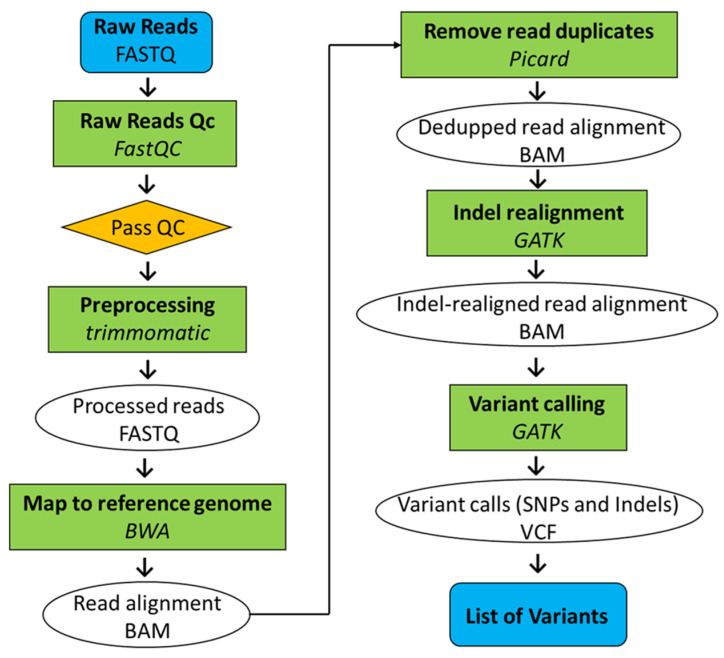
Pipeline of whole genome resequencing analysis.

Whole genome sequencing was conducted on both Koshishikari Hd16 (BC_7_F_4_) and Koshihkari sd1Hd16 (BC_8_F_2_), which were integrated with the late flowering gene *Hd16* and semidwarfing gene *sd1*, by eight backcrosses into the genetic background of Koshihikari. The leaves were powdered using a mortar and pestle, and frozen in liquid nitrogen. Genomic DNA was extracted from each cultivar using the cetyltrimethylammonium bromide method. Genomic DNA was fragmented and simultaneously tagged so that the peak size of the fragments was approximately 500 bp using the Nextera^®^ transposome (Illumina Inc., San Diego, CA, USA). After purification of the transposome using DNA Clean & ConcentratorTM-5 (Zymo Research, Irvine, CA, USA), adaptor sequences, including the sequencing primers, for fixation on the flow cell were synthesized at both ends of each fragment using polymerase chain reaction. The DNA fragments were then subjected to size selection using AMPure XP magnetic beads (Beckman Coulter, Brea, CA, USA). Finally, qualitative checks were performed using a Fragment Analyzer™ (Advanced Analytical Technologies, Heidelberg, Germany) and quantitative measurements using Qubit^®^ 2.0 Fluorometer (Life Technologies; Thermo Fisher Scientific, Inc., Waltham, MA, USA) to prepare a DNA library for next-generation sequencing. Sequencing was conducted in paired-end 2 × 100 bp on a HiSeq X next-generation sequencer, according to the manufacturer’s protocol (Illumina Inc., San Diego, CA, USA). Illumina reads were trimmed using Trimmomatic (version 0.39) [40] (Figure 3). Sequencing adapters and sequences with low quality scores on the 3′ ends (Phred score [Q], <20) were trimmed. The raw Illumina whole genome sequence reads were quality checked by performing quality control using FastQC (version 0.11.9; Babraham Institute, Cambridge, UK). Mapping of reads from Koshihikari Hd16 and Koshishikri sd1Hd16 to the Koshishikri genome as a reference was conducted using Burrows-Wheeler Aligner software (version bwa-0.7.17.tar.bz2; Appirits, Tokyo, Japan) [41]. Duplicated reads were removed using Picard (version 2.25.5; GitHub Inc., CA, USA) and secondary aligned reads were removed using SAMtools (version 1.10.2; SourceForge, CA, USA) [42]. To identify genetic variations among strains, single nucleotide variant detection (variant calling) and single nucleotide variant matrix generation were performed using GATK (version 4.1.7.0; Broad Institute, Cambridge, MA, USA) [43].

## 3. Results

### 3.1. Development of Semidwarf Late-Maturing Koshihikari-Type Isogenic Line Koshihikari sd1Hd16

**Figure 4:**
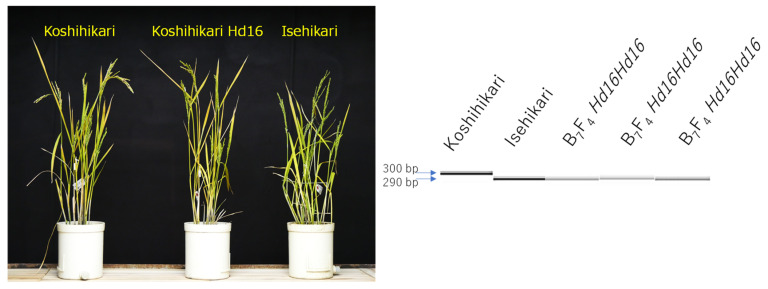
Plant type of Koshihikari Hd16 (BC_7_F_4_) screened by diagnosis for *Hd16* by single sequence repeat marker RM16089. *Hd16* homozygous plant (BC_7_F_4_) was identified by gene diagnosis using SSR marker RM16089.

**Figure 5:**
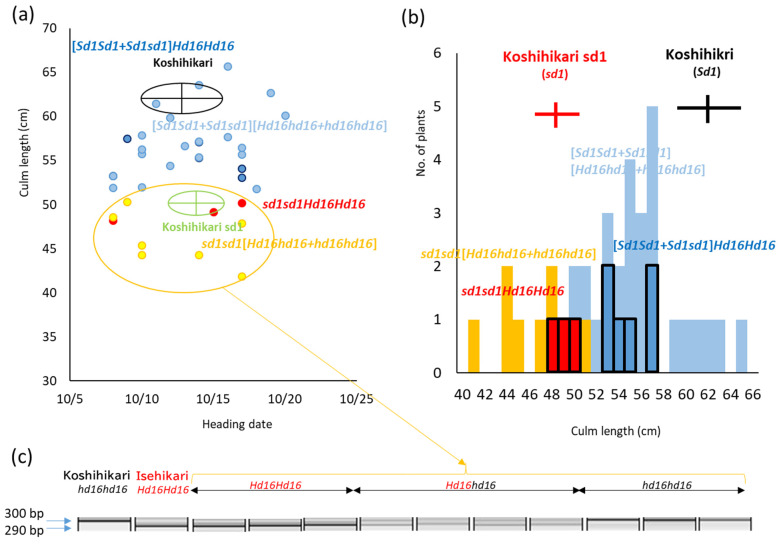
Phenotypic segregation for the *sd1* allele based on culm length and genotypic segregation of the *Hd16* allele based on genetic diagnosis in BC_7_F_2_ of Koshihikari sd1 × Koshihikari *Hd16* (BC_6_F_2_). (**a**,**b**) In Koshihikari *sd1* × Koshihikari *Hd16* (BC_6_F_2_), F_2_ plants were segregated in a ratio of 26 long-culm type [51–66 cm, similar to Koshihikari]:10 semidwarf type [41–51 cm, similar to Koshihikari sd1 with slightly darker green color] ≈ 3 [*Sd1* homozygous + heterozygous]:1 [*sd1* homozygous] (χ^2^ = 0.14, 0.65 < *p* < 0.70) in the *sd1* allele, according to single gene segregation. (**c**) Furthermore, 10 semidwarf plants were segregated in a ratio of 3 Isehikari homozygous (*Hd16* homozygous): 4 heterozygous: 3 Koshihikari type (*hd16* homozygous) by genetic diagnosis using the nearby single sequence repeat marker RM16089.

In the present study, *Hd16* homozygous plant (BC_7_F_4_) was identified by gene diagnosis using SSR marker RM16089 (Figure 4). Next, in Koshihikari *sd1* × Koshihikari *Hd16*, BC_7_F_2_ plants were segregated in a ratio of 26 long culm types [51–66 cm, similar to Koshihikari]:10 semidwarf types [41–51 cm, similar to Koshihikari sd1 with slightly darker green color] ≈ 3 [*Sd1* homozygous + heterozygous]:1 [*sd1* homozygous] (χ^2^ = 0.14, 0.65 < *p* < 0.70) in the *sd1* allele, according to single gene segregation (Figure 5a,b). In contrast, regarding the *Hd16* allele, as a result of genetic diagnosis using the nearby SSR marker RM16089, F_2_ plants were segregated in a ratio of 9 Isehikari homozygous (*Hd16* homozygous): 15 heterozygous: 12 Koshihikari type (*Hd16* homozygous) ≈ 1:2:1 (χ^2^ = 1.50, 0.45 < *p* < 0.50). Furthermore, regarding the *sd1* allele, 9 *Hd16* homozygous plants were segregated into 6 long-culm types [*Sd1* homozygous + heterozygous]:3 semidwarf types [*sd1* homozygous] ≈ 3:1 (χ^2^ = 0.33, 0.55 < *p* < 0.60), 15 *Hd16Hd16* heterozygous plants were segregated into 11 long-culm types: 4 semidwarf type ≈ 3:1 (χ^2^ = 0.022, 0.95 < *p* < 0.99), and 12 *Hd16* homozygous plants were segregated into 9 long-culm types: 3 semidwarf type ≈ 3:1 (χ^2^ = 0.00, 0.95 < *p* < 0.99). That is, as a whole, F_2_ plants were segregated into 20 long-culm/early maturing plants: 6 long-culm/late-maturing plants: 7 semidwarf/early maturing plants: 3 semidwarf/late-maturing plants ≈ 9 (*Sd1Sd1Hd16Hd16* + 2*Sd1Sd1Hd16Hd16* + 2*Sd1sd1Hd16Hd16* + 4*Sd1sd1Hd16Hd16*): 3 (*Sd1Sd1Hd16Hd16* + 2*Sd1sd1Hd16Hd16*): 3 (*sd1sd1Hd16Hd16* + 2*sd1sd1Hd16Hd16*): 1 (*sd1sd1Hd16Hd16*) (χ^2^ = 0.35, 0.90 < *p* < 0.95) according to two-gene segregation. Therefore, *sd1* and *Hd16* were independently inherited. Thus, a semidwarf late-maturing Koshihikari-type isogenic line Koshihikari sd1Hd16 having *sd1* and *Hd16* homozygous was developed.

**Figure 6:**
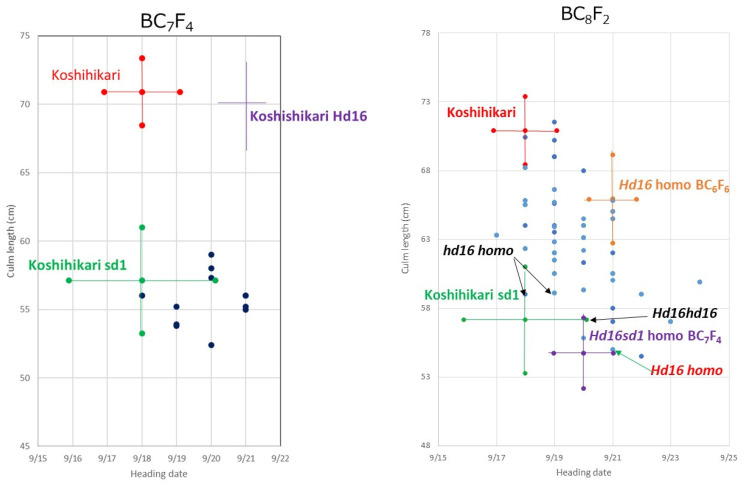
Relationship between heading date and culm length in Koshihikari/Koshihikari sd1/Koshihikari × 6/[(Koshihikari × Isehikari) F_2_] BC_8_F_2_ and BC_7_F_4_ progeny of its parent. In BC_8_F_2_, *sd1* homozygous plants, whose culm length were 54.5–59.1 cm, similar to that of Koshihikari sd1 with short, thick and dark green flag leaves, intermediate type plants, and the *Sd1* homozygous plants whose culm length were 65.6–71.5 cm, similar to that of Koshihikari with long and thin color flag leaves, were segregated in a ratio of 10:25]:12 ≈ a single gene inheritance theoretical ratio of 1 [*sd1* homozygous]:2 [intermediate type: 1 [*Sd1* homozygous] (χ^2^ = 0.36, 0.80 < *p* < 0.90).

**Figure 7:**
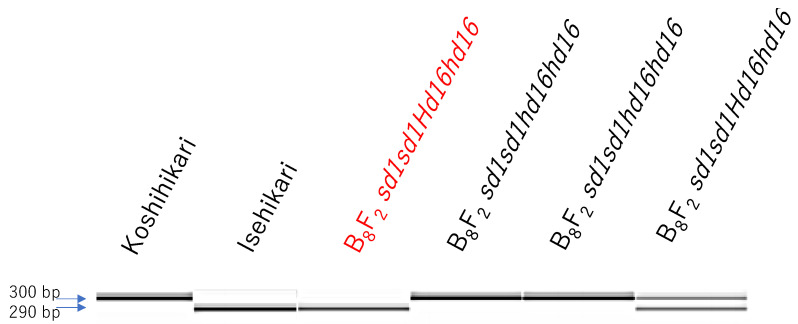
Genetic diagnosis for *Hd16* allele using the single sequence repeat marker RM16089 in BC_8_F_2_. From BC_8_F_2_, four *sd1* homozygous plants were arbitrarily selected based on culm length and genetically diagnosed using RM16089. One plant was *Hd16* homozygous, one was heterozygous, and two were *hd16* homozygous.

**Figure 8:**
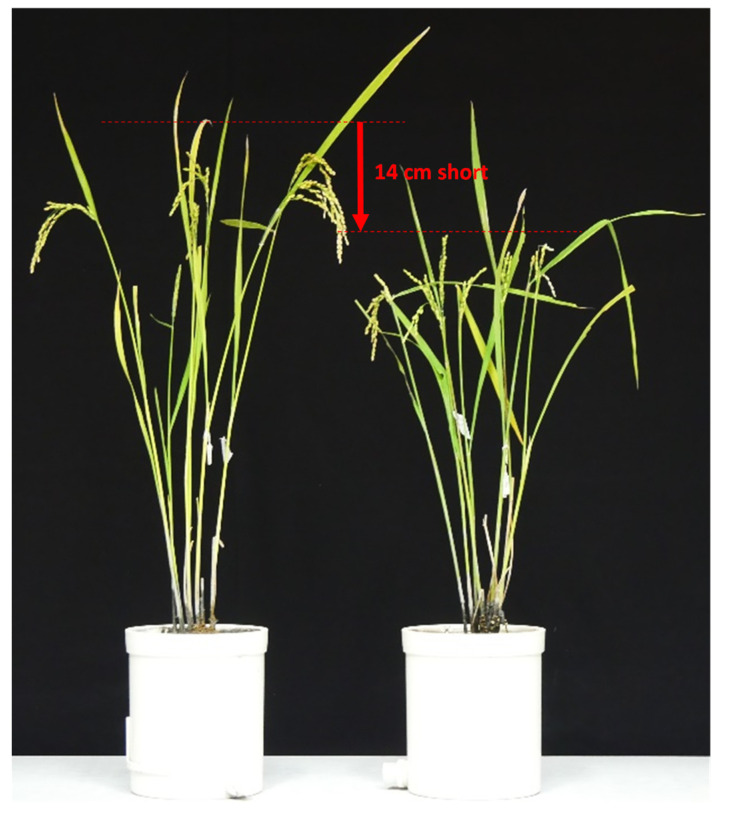
Morphological alteration of Koshihikari sd1Hd16 (BC_8_F_2_) that integrates *sd1* and *Hd16* in the genetic background of Koshihikari. The semidwarf late-maturing Koshihikari isogenic line having *sd1* and *Hd16* homozygous acquired in BC_8_F_2_ was 14.2 cm (15%) shorter than Koshihikari and was characterized by a deep green color.

The relationship between culm length and heading date in BC_8_F_2_, in which the *Hd16Hd16Sd1sd1* plant segregated in BC_7_F_2_ was crossed as the pollen parent with Koshihikari as the mother, is shown in Figure 6. In BC_8_F_2_, *sd1* homozygous plants, whose culm lengths were 54.5–59.1 cm, similar to that of Koshihikari sd1 with short, thick and dark green flag leaves, intermediate plants, and the *Sd1* homozygous plants whose culm lengths were 65.6–71.5 cm, similar to that of Koshihikari with long and thin color flag leaves, were segregated in a ratio of 10:25:12 ≈ a single gene inheritance theoretical ratio of 1 [*sd1* homozygous]:2 [intermediate type]:1 [*Sd1* homozygous] (χ^2^ = 0.36, 0.80 < *p* < 0.90). From BC_8_F_2_, four *sd1* homozygous plants were arbitrarily selected based on culm length and genetically diagnosed using RM16089. One plant was *Hd16* homozygous, one was heterozygous, and two were *hd16* homozygous (Figure 7). The semidwarf late-maturing Koshihikari isogenic line with *sd1* and *Hd16* homozygotes was acquired in BC_8_F_2._ Koshihikari sd1Hd16 flowered 12.1 days later than Koshishikari or Koshihikari sd1 did, and was 14.2 cm (15%) shorter than Koshihikari, with a characteristic deep green color (Figure 8). The yield of Koshishikari sd1Hd16 (63.2 kg/a) was 7.0% higher than that of Koshihikari (Table 1).

### 3.2. Whole Genome Sequencing of Koshihikari sd1Hd16

**Figure 9:**
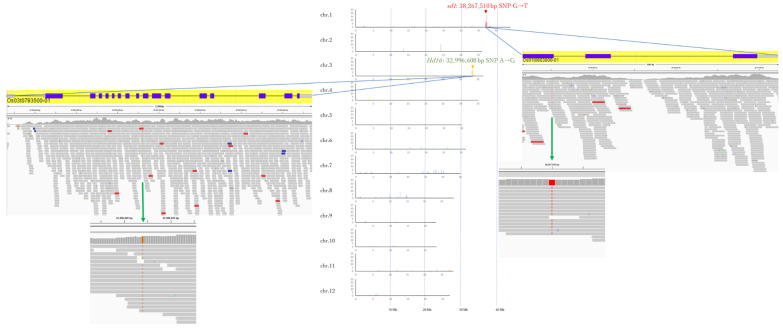
Causative SNP for Hd16 in Koshihikari sd1Hd16 (BC_8_F_2_). In both Koshihikari Hd16 (BC_7_F_4_) and Koshihikari sd1Hd16 (BC_8_F_2_), SNPs from adenine to guanine were detected in *Hd16* at 32,996,608 bp on chromosome 3, which is known to be a causative mutation of Hd16 in Nipponbare. In Koshihikari sd1Hd16 (BC_8_F_2_), SNPs from thymine to guanine were detected in *sd1* at 38,267,510 bp on chromosome 1.

The number of reads decoded by the next-generation sequencer was 41,630,793 for Koshihikari sd1Hd16 (BC_8_F_2_). The obtained reads of Koshishikri sd1GW2 were mapped to the consensus sequence of Koshihikari as a reference, and the mean coverage was 23.72. After removing the secondary alignment and duplicate reads, the unique reads were 31,249,311. A total of 43,861 SNPs [homozygous 2044, heterozygous 41,817] were detected. In both Koshihikari Hd16 (BC_7_F_4_) and Koshihikari sd1Hd16 (BC_8_F_2_), SNPs from adenine to guanine were detected in *Hd16* at 32,996,608 bp on chromosome 3, which is known to be a causative mutation of *Hd16* in Nipponbare. In Koshihikari sd1Hd16 (BC_8_F_2_), SNPs from thymine to guanine were detected in sd1 at 38,267,510 bp on chromosome 1. Except for the region around *Hd16* and *sd1*, the number of SNPs was less than 10 per 0.1 Mb. The results indicated that a large portion of the 12 chromosomes in rice was substituted into the genome of Koshihikari (Figure 9) after continuous backcross targeting of these two genes.

**Figure 10:**
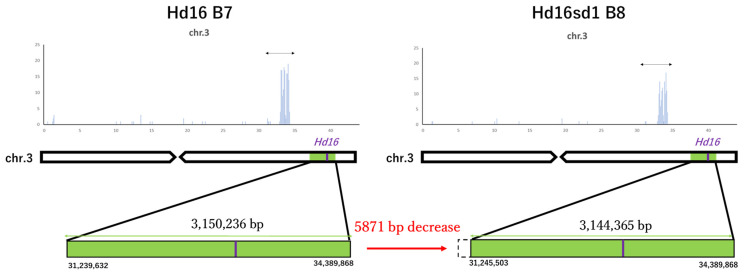
Transitional change in the size of DNA fragment integrated with *Hd16* via backcrossing. The size of the DNA fragment integrated into *Hd16* was determined as the distance between both ends of an SNP cluster. After a single backcross from BC_7_ to BC_8_, the size of the DNA fragment integrated with *Hd16* decreased by 5871 bp.

After a single backcross, the total number of SNPs decreased from 725 in Koshihikari Hd16 (BC_6_F_2_) to 348 in BC_7_. The size of the DNA fragment integrated into Hd16 was determined as the distance between both ends of an SNP cluster. In Koshihikari Hd16 (BC_6_F_2_), it was 3,150,236 bp, from 31,239,632 bp to 34,389,868 bp in the short arm of chromosome 3. In contrast, in Koshihikari sd1Hd16 (BC_7_F_4_), it was 3,144,365 bp, from 31,239,632 to 34,389,868 bp. Therefore, after one backcross from BC_6_ to BC_7_, the size of the DNA fragment integrated with *Hd16* decreased by 5871 bp (Figure 10). A total of 617 annotated genes were identified in the integrated DNA fragments. Among them, there were mutations in four genes, including the zinc finger protein gene (Table 2).

## 4. Discussion

The threat of strong typhoons, rainfall, and floods caused by global warming causes serious lodging [44], resulting in yield loss and grain quality deterioration in rice production [2]. The first author developed Koshihikari sd1, designated as Hikarishinseiki [21,23], and registered it under the Plant Variety Protection Act in Japan and the United States [23,25]. Koshihikari sd1 was approximately 20 cm shorter than Koshihikari, and its genome consists of more than 99.8% of the genome of Koshihikari, except for *sd1* derived from Jukkoku [20,21]. However, Koshihikari also suffers from poor filling and yield reduction, caused by high-temperature maturation. To avoid high-temperature damage in the hot summer, shifting rice ripening to early autumn is an effective solution. In this study, first, the late-maturing gene *Hd16* from Isehikari was integrated into Koshishikari by seven backcrosses with Koshihikari as the recurrent parent using a late-maturing plant as a non-recurrent parent that was segregated in F_2_ of Koshihikari × Isehikari. Then, the late maturing isogenic Koshishikri Hd16 was crossed with Koshihikari sd1 to combine the semidwarf gene *sd1* and *Hd16* into the genetic background of Koshihikari, and eight backcrosses to the genetic background of Koshihikari were completed to build isogenic Koshihikari integrating both *Hd16* and *sd1*. Through the backcross process, *Hd16* allele was diagnosed by SSR marker RM16089 near the *Hd16* allele, and *sd1* homozygotes were successfully selected by their phenotype in each relativity-limited BCnF_2_ population. Finally, whole genome sequencing of Koshihikari sd1Hd16 showed that an SNP from adenine to guanine was detected at 32,996,608 bp in *Hd16* on chromosome 3, and an SNP from thymine to guanine was detected in *sd1* at 38,267,510 bp on chromosome 1. The size of the DNA fragment integrated with *Hd16* was determined to be 3,144,365 bp in Koshihikari Hd16sd1, based on the distance between both ends of an SNP cluster. After backcrossing BC_7_ to BC_8_, the size of the DNA fragment integrated with *Hd16* decreased by 5871 bp.

The SNP found in *Hd16* from Isehikari was the same as that found in Nipponbare. *Hd16* of Nipponbare encodes casein kinase I [45,46]. Under long-day conditions, *Hd16* acts upstream of the photosensitive floral repressor gene *Ghd7* and phosphorylates the transcript of *Ghd7*, which is located upstream of the flowering gene *Ehd1*, to strengthen photosensitivity and delay flowering [46,47]. The introgression of *Hd16* from Nipponbare into Koshihikari has been reported to cause a 10-day delay in maturation [46,48,49,50]. In the present study, we developed an isogenic line via eight backcrosses and clarified the genome structure in which almost all sequences were replaced by the Koshihikari genome, except for the vicinity of *Hd16* on chromosome 3 derived from Isehikari and *sd1* derived from Jukkoku on chromosome 1. In the present study, the same nonsynonymous substituted *Hd16* allele from Isehikari resulted in decreased photoperiod sensitivity to noticeably delay flowering time by 12 days, which was considered to be attained in the highly isogenic background. Twelve days of late-flowering Koshihikari owing to *Hd16* will avoid flowering and ripening during the high-temperature period in the hottest summer period in August. Furthermore, it is one of the promising options of a regionally adaptive genotype to address the overuse of Koshihikari throughout Japan. Late-maturing Koshihikari is highly desired in the rice industry. The yield merit underpinning *Hd16* has been previously reported [51]. Ministry of Agriculture, Forestry and Fisheriesof Japan (MAFF) has registered the late-maturing isogenic Koshihikari, which was integrated with *Hd16,* designated as a new plant variety ’Koshihikari Suruga Hd16′ [52] under Japanese varietal protection. Furthermore, the author has applied for Japanese varietal protection for the late-maturing and semidwarf isogenic Koshihikari, which was integrated with both *Hd16* and semidwarf gene *sd1,* designated as a new plant variety ’Koshihikari Suruga sd1Hd16′ [53]. This is a new germplasm designed to avoid heat damage at ripening during high-temperature summer periods by late maturation owing to *Hd16* as well as to avoid lodging by autumn typhoons by semidwarfness owing to *sd1*.

## 5. Conclusions

We developed a semidwarf and late-maturing isogenic Koshihikari sd1Hd16 (BC_8_F_2_) strain to stabilize high yield and avoid high-temperature maturation. Whole genome analysis detected an SNP from adenine to guanine in *Hd16* at 32,996,608 bp on chromosome 3, and an SNP from thymine to guanine was detected in *sd1* at 38,267,510 bp on chromosome 1. From BC_7_ to BC_8_, the size of the DNA fragment integrated with *Hd16* decreased by 5871 bp. Koshihikari sd1Hd16 flowered 12.1 days later than Koshishikari or Koshihikari sd1 did and was 14.2 cm (15%) shorter than Koshihikari. Koshishikari sd1Hd16, with a 7.0% higher yield than Koshihikari, is a new germplasm to avoid heat damage during ripening during high-temperature summer by *Hd16* as well as to avoid lodging by autumn typhoons by *sd1*.

## Figures and Tables

**Table 1:** Comparison of agronomic characters of Koshihikari, Koshishikri Hd16, Koshishikari sd1, and Koshihikari sd1Hd16.

Genotypes	Days to Heading	Culm Length (cm)	Panicle Length (cm)	No. of Panicles (No./m^2^)	1000-Grain Weight (g)	Grain Yield (kg/a)	Lodging Degree	Protein Content	Value of Taste
Koshihikari	76.8	92.6	18.9	359	22.6	59.1	3.2	6.8	0.00
Koshihikari Hd16	88.3 *	109.0 *	19.6	320 *	22.6	61.	3.5	6.8	−0.13
Koshihikari sd1	77.0	74.0 *	18.8	375 *	22.8	60.8	0.0 *	7.1	0.14
Koshihikari sd1Hd16	88.9 *	78.4 *	19.1	398 *	23.0	63.2 *	0.0 *	7.0	−0.06

Koshihikari sd1Hd16 flowered 12.1 days later than Koshishikari or Koshihikari sd1 did and was 14.2 cm (15%) shorter than Koshihikari. The yield of Koshishikari sd1Hd16 (63.2 kg/a) was 7.0% higher than that of Koshihikari. *: statistically significant at the 5% level.

**Table 2:** Mutant genes integrated with *Hd16* via backcrossing.

Position (bp)	Gene Name	Description
33,625,002	Os03g0805400	Phosphatidic acid phosphatase type 2/haloperoxidase domain containing protein
33,687,624	Os03g0806400	Elongation factor P family protein
34,032,614	Os03g0812200	Zinc finger, RING/FYVE/PHD-type-domain containing protein.
34,055,448	Os03g0812800	Calcium-binding allergen Ole e 8-like protein

A total of 617 annotated genes were identified in the integrated DNA fragments. Among them, there were mutations in four genes, including the zinc finger protein gene.

## Data Availability

All the data generated in this study are present in the main manuscript.
